# Joule-Heating Effect of Thin Films with Carbon-Based Nanomaterials

**DOI:** 10.3390/ma15124323

**Published:** 2022-06-18

**Authors:** Usha Kiran Sanivada, Dina Esteves, Luisa M. Arruda, Carla A. Silva, Inês P. Moreira, Raul Fangueiro

**Affiliations:** 1 Fibrenamics—Institute of Innovation in Fiber-Based Materials and Composites, Azurém Campus, 4800-058 Guimarães, Portugal; dinaesteves81@gmail.com (D.E.); luisa.arruda@fibrenamics.com (L.M.A.); ines.moreira@fibrenamics.com (I.P.M.); 2 Mechanical Engineering and Resources Sustainability Centre (MEtRICS), Azurém Campus, University of Minho, 4800-058 Guimarães, Portugal; 3 Centre for Textile Science and Technology (2C2T), Azurém Campus, University of Minho, 4800-058 Guimarães, Portugal; 4 Simoldes Plastics, Research & Innovation, Rua Comendador António da Silva Rodrigues 165, 3720-502 Oliveira de Azeméis, Portugal; carla.a.silva@simoldes.com

**Keywords:** smart textiles, nanomaterials, joule heating, screen printing, electrical conductivity, resistance

## Abstract

Smart textiles have become a promising area of research for heating applications. Coatings with nanomaterials allow the introduction of different functionalities, enabling doped textiles to be used in sensing and heating applications. These coatings were made on a piece of woven cotton fabric through screen printing, with a different number of layers. To prepare the paste, nanomaterials such as graphene nanoplatelets (GNPs) and multiwall carbon nanotubes (CNTs) were added to a polyurethane-based polymeric resin, in various concentrations. The electrical conductivity of the obtained samples was measured and the heat-dissipating capabilities assessed. The results showed that coatings have induced electrical conductivity and heating capabilities. The highest electrical conductivity of (9.39 ± 1.28 × 10^−1^ S/m) and (9.02 ± 6.62 × 10^−2^ S/m) was observed for 12% (*w*/*v*) GNPs and 5% (*w*/*v*) (CNTs + GNPs), respectively. The sample with 5% (*w*/*v*) (CNTs + GNPs) and 12% (*w*/*v*) GNPs exhibited a Joule effect when a voltage of 12 V was applied for 5 min, and a maximum temperature of 42.7 °C and 40.4 °C were achieved, respectively. It can be concluded that higher concentrations of GNPs can be replaced by adding CNTs, still achieving nearly the same performance. These coated textiles can potentially find applications in the area of heating, sensing, and biomedical applications.

## 1. Introduction

Even though textiles have historically mainly been used for clothing purposes, the advancement of technology has opened up their use for various other applications. Textile materials possess high electrical resistance, low electrical conductivity, and low thermal conductivity in their pristine state [1]. Developments in nanotechnology have given the opportunity of introducing new functionalities to textiles, endowing the production of smart and intelligent textiles [2]. The functionalization of textiles with nanomaterials can introduce value-added functionalities such as electrical conductivity, thermal conductivity, sensing capabilities, antimicrobial properties, and ultraviolet light protection, among others. These can be integrated into many applications such as sports, protection, healthcare, fashion, transportation, and automobiles [3]. Depending on the main focus, the challenge is sometimes to produce functional textiles while keeping their inherent properties. One of the critical requirements for being a smart textile is having electrical conductivity besides being flexible and lightweight [4]. Light weight, high stretchability, and good deformation flexibility are the vital factors for textiles to be used for flexible devices, with conductive textiles standing as a good fit for these applications [5]. Flexibility, stretchability, and mechanical stability of textiles are considered crucial parameters for wearable electronics, as these are subjected to several mechanical deformations upon usage [6]. Washability might be another one of the essential features for wearable electronics if they need to keep their properties after several washes [7]. However, the focus of this paper is on textiles with improved electrical and thermal conductivities for heating applications. Joule law explains the dissipation of energy in the form of heat once a current is applied to a certain material. A very conductive material will not present any resistance to the passage of current, which is why a conductive material should have some resistivity value to be able to produce heat [1].

Cotton textiles have low conductivity and high resistance value in their pristine state and are considered low-conductive materials [4]. From the literature, it can be noted that electrical conductivity can be enhanced in two ways: by introducing the yarn with conductive properties in the fabrics and by coating it with conductive materials [8]. Among many techniques such as coating, exhaustion, padding, dip dry, and so forth, coating by screen printing is one of the easiest methods that can be used to modify the properties of the textile. It includes the coating of a polymeric matrix onto the surface of the substrate either on one side or two sides, followed by curing. Different nanomaterials can be added to the paste to exploit any additional properties [2]. Coating the textiles with the conductive paste will facilitate the transfer of electrons. This type of method is gathering attention as it can produce metal-free smart textiles that are flexible [9].

Carbon-based materials such as CNTs [5,10], graphene-related materials such as graphene oxide [11], reduced graphene oxide [12,13], GNPs [14], and other nanomaterials such as silver and copper nanoparticles or conductive polymers have been introduced into textiles to improve their conductivity and endow them with heat-dissipating capabilities [15]. Moreover, the addition of carbon-based nanofillers in the polymer matrix will improve lubrication action [16]. Graphene is one of the most popular carbon nanofillers, consisting of a planar sheet of one-atom thickness and carbon atoms with sp^2^ hybridization in the form of a honeycomb lattice. It has different forms such as graphene nanolayers, graphene nanosheets, and GNPs. Graphene has good mechanical, thermal, and electrical properties, which are shown in Table 1 [17]. Despite having good properties, it is not feasible to produce graphene on an industrial scale due to the high cost associated with its production. Hence, researchers are looking towards GNPs as an alternative to graphene, where they can be produced by ball milling, shear exfoliation, etc. GNPs are attractive as they can be mixed into the polymeric matrix to produce coatings or nanocomposites, and their electrical conductivity is in the order of 6 × 10^3^ S/m [18]. Coating of these nanomaterials can be performed via screen printing, which is one of the effective coating techniques alongside pad printing, flexography printing, gravure printing, and roll-to-roll printing. Screen printing is more often preferred because it can produce layers of thickness in the range of 5–25 µm, prints can be reproduced easily, and different designs can be obtained. Not only it is cost-effective, but it is also scalable to the industrial process [2].

Cotton fabrics coated with polyurethane mixed with GNPs were investigated for electrical resistivity, and it was observed that the resistivity reduced to a magnitude of 8 orders [19]. Likewise, in other research works [20,21], graphene was used as filler in polyurethane-based polymers and natural rubber latex [22], with enhanced electrical conductivity of the fabrics. Interestingly, besides cotton fabrics, cotton in the fiber form was mixed with polydimethylsiloxane (PDMS) along with multiwalled carbon nanotubes (MWCNTs) to create a conductive composite [23]. Similarly, some studies [24,25] used a combination of CNTs and GNPs to improve electrical conductivity.

The main objective of this study was to introduce a specific range of electrical conductivity to the pristine cotton fabrics by coating different layers of paste made of a polyurethane-based resin with dispersed GNPs and CNTs. The coated samples were characterized by Raman spectroscopy, thermogravimetric analysis (TGA), and field-emission scanning electron microscopy (FESEM). They were also investigated for their electrical conductivity and heat-dissipation capabilities.

## 2. Materials and Methods

### 2.1. Materials

Polyurethane-based resins and thickener, commercially available as Edolan, Tanapur, and Thickener from Tanatex Chemicals, Netherlands, were used in this work. GNPs and MWCNTs were obtained from Graphenest, Portugal and Iolitec Ionic Technologies, Germany. The cotton fabric was purchased from Lameirinho, Portugal. The technical data of the materials are presented in Table S1 (Supplementary Materials). The paste for coating was prepared by mixing the polymer and GNPs and CNTs in various weight fractions using a mechanical mixer. Screen printing was used to produce cotton-coated samples with several layers.

### 2.2. Paste Preparation

**Figure 1:**
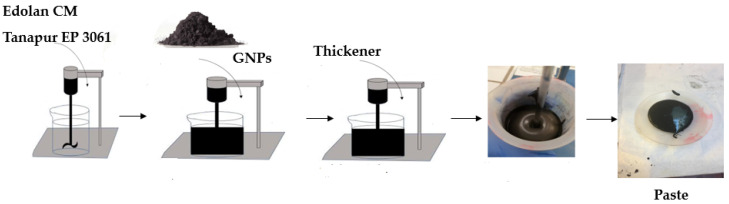
Schematic representation of the preparation of paste with polymer and carbon-based nanomaterials.

The paste was prepared by mixing the polymer resin with the graphene nanoplatelets (procedure represented in Figure 1). Initially, the polymer Edolan CM and Tanapur EP30651 (50:50) were stirred for 10 min with the help of a mechanical mixer at 350 rotations per minute. Later on, GNPs were added to the polymer and stirred for 30 min. Then, different proportions (~0.02 to 0.07% *w*/*v*) of the thickener were added and subjected to mixing for 30 min to obtain the desired viscosity for the paste. GNPs were added to the polymer in such a way that 2% (*w*/*v*), 3% (*w*/*v*), 5% (*w*/*v*), 7% (*w*/*v*), 10% (*w*/*v*), and 12% (*w*/*v*) concentrations were obtained. Following a similar procedure, later the paste with a combination of 5% (*w*/*v*) (GNPs and CNTs) in the ratio of 50:50 i.e., 2.5% (*w*/*v*) of each was prepared where CNTs were added to the polymer initially, then GNPs, and followed by mixing the paste for 30 min.

**Figure 2:**
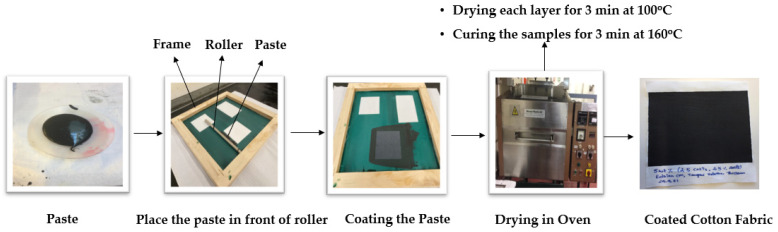
Steps involved in the screen-printing process.

The obtained paste was coated on the cotton substrate using the screen-printing method. The samples were coated with 1, 2, and 3 layers for each concentration using screen printing, as shown in Figure 2. A Johannes Zimmer table, Austria, and a frame produced in our lab having a polyester mesh (Weft density = 34/cm^2^, Warp density = 31/cm^2^) were used to perform the screen-printing process. The cotton fabric was placed below the frame and the roller was moved by placing the paste in front of the roller to obtain coatings on the cotton fabric, the sample was dried for 3 min at 100 °C, and cured for 3 min at 160 °C in the oven (Mathis AG, Oberhasli and Switzerland).

### 2.3. Characterization Techniques

#### 2.3.1. Film Thickness

The thickness of the coated fabrics was measured using a digital caliper from FISCHER DAREX. The instrument can measure a thickness of ~0.01 mm. The thickness of the layer is given by the difference in thickness of the fabric with coating and without coating. An average of ten measurements were taken for the thickness of all the samples.

#### 2.3.2. Raman Spectroscopy

Raman spectra of the samples that contain carbon-based coatings were attained from Horiba LAbRAM HR Evilution confocal microscope (Horiba Scientific, Longjumeau, France) and it is equipped with a 532 nm (2.33 eV) laser. The samples were placed on the glass side and the area of the samples are 40 × 20 mm^2^. A focused laser on the samples with the help of a 100× objective lens was used to investigate the structure and quality of the GNPs and CNTs. The one-layer coated samples produced with 2%, 3%, 5%, 7%, 10, 12% (*w*/*v*) GNPs and 5% (*w*/*v*) (CNTs + GNPs) were analyzed. For each sample, at least four scans were taken at places chosen randomly to ensure homogenous analysis. LabSpec 6 software was used for analyzing the results. The intensity ratio of I_D_/I_G_ was calculated to identify the quality of the nanomaterial crystalline structures.

#### 2.3.3. Thermogravimetric Analysis

The thermal stability of the samples was determined by thermogravimetric analysis (TGA) using SDT-2960 (TA Instruments). The TGA analysis was performed over a temperature range of 30–600 °C with a heating rate of 20 °C/min in an argon gas environment. The samples coated with 5% (*w*/*v*) GNPs, 5% (*w*/*v*) (CNTs + GNPs), and 12% (*w*/*v*) GNPs one layer besides pristine cotton and coated with polymers and thickener were selected to perform TGA.

#### 2.3.4. Field-Emission Scanning Electron Microscopy

The presence of GNPs and their dispersion in the coated samples were studied by the Field-Emission Scanning Electron Microscopy (FESEM) images. The morphology of the coated surface and the cross-sections of the samples were examined using the NOVA 200 Nano SEM machine manufactured by the FEI company (Hillsboro, OR, USA). A palladium-gold film of 20 nm was sputter-coated on the samples before performing the analysis.

#### 2.3.5. Electrical Conductivity

**Figure 3:**
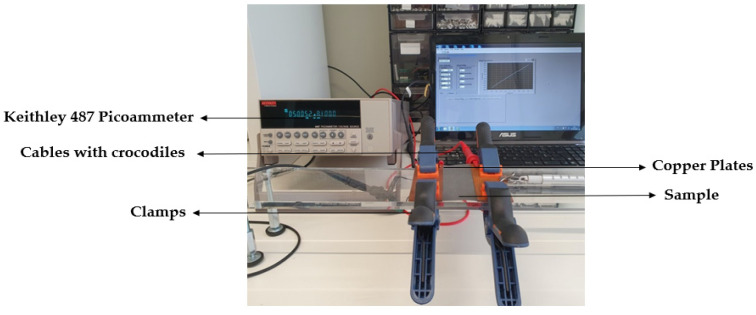
Setup for measuring electrical resistance.

The electrical conductivity of the produced samples was measured by using a Keithley 487 Picoammeter/voltage source. Clamps and copper plates were used to fix the samples and connect them to the setup as shown in Figure 3. In this method, the current passing through the sample was measured by sending the voltage between −0.8 V to 0.8 V with a step of 0.1 V across the sample. An average of five readings was taken and the graph was drawn between voltage and current, where the slope of the graph gives the value of resistance of the sample. After obtaining the resistance R in Ω, electrical resistivity can be calculated by using the equation below [18]. Once the resistivity (ρ) in Ω m was obtained, the inverse of resistivity gave the conductivity (σ) in (S/m).

(1) $$\rho =R*\;\frac{A}{L}(Ωm)$$

where

ρ = Resistivity

*R* = Resistance (Ω)

*A* = Cross-sectional area (m^2^)

*L* = Distance between the electrodes (m)

#### 2.3.6. Joule Heating

**Figure 4:**
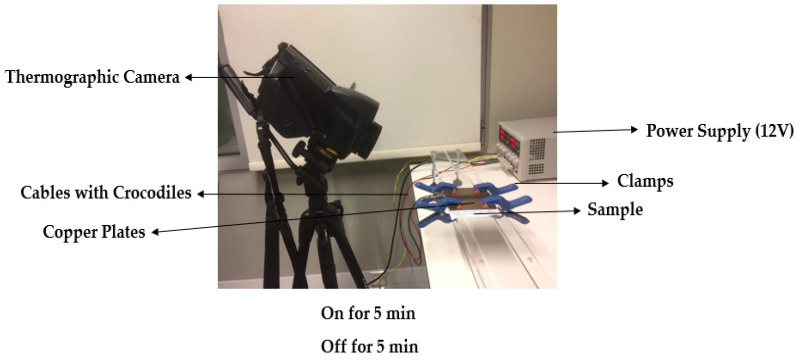
Setup for measuring joule heating.

The setup used to measure the joule heating consisted of a CPX200 D Dual 180-Watt DC supply source from Power Flex with crocodile cables, four copper plates, clamps, acrylic, and a thermal imager (Testo 885) from TESTO, as shown in Figure 4. The sample was fixed in between the copper plates and they were attached to the DC supply source with the crocodile cables. A voltage of 12 V was applied to the coated cotton textile with the help of a DC supplier. The voltage was applied for 300 s and the rise in temperature at different locations was measured by using a thermographic camera followed by measuring the fall in temperature for 300 s after stopping the voltage from the supply, at regular intervals of 30 s. The temperatures in heating mode and cooling mode for 300 s each were registered and the graphs were drawn for the time and temperature. The maximum temperature attained after supplying 12 V for 300 s was recorded for the samples.

## 3. Results and Discussions

### 3.1. Film Thickness

The thicknesses of the films were measured (Table 2) and they were used to calculate the resistivity and conductivity of the coated samples. The thickness of the samples increased with the increase in the layers of coatings and slightly with the increase in the nanofillers’ concentration. The thickness of the films influences their respective resistivities and conductivities. The resistivity decreases and conductivity increases with thickness. However, above a certain value of thickness, the carrier mobility decreases and the conductivity will be reduced [26].

### 3.2. Raman Spectroscopy

**Figure 5:**
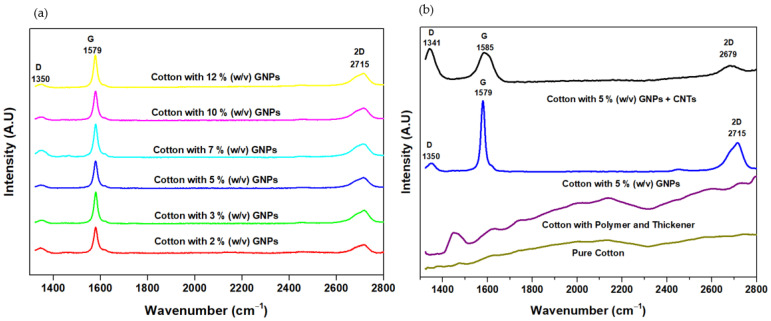
Raman spectra of various samples (**a**). Composition of GNPs varying from 2% (*w*/*v*), 3% (*w*/*v*), 5% (*w*/*v*), 7% (*w*/*v*), 10% (*w*/*v*) and 12% (*w*/*v*); (**b**). Pure cotton, cotton with polymer and thickener, 5% (*w*/*v*) GNPs and 5% (*w*/*v*) (GNPs + CNTs).

Raman spectroscopy is one of the best noninvasive techniques to characterize carbon-based materials such as GNPs and CNTs [27]. The allotropy forms of carbon are made up of carbon-carbon bonds; however, the hybridization of these bonds can be different. The modifications of these C-C bonds can be detected effectively by Raman spectroscopy [28]. The D and G band peaks represent the disordered sp^3^ and sp^2^ graphite structures, respectively [29]. Figure 5a,b represents D, G, and 2D bands of GNPs and (GNPs + CNTs). The peak at 1350 cm^−1^ represents the D band and corresponds to the A_1g_ mode, which represents vibration modes of dangling bonds of disordered GNPs [30]. Similarly, the peak at 1579 cm^−1^ corresponds to the G band, which represents the E_2g_ mode, and it can imply the vibration of the sp^2^ bonded carbon atoms [30]. Eventually, the peak at 2715 cm^−1^ can be correlated to 2D and is obtained due to the sp^2^ hybridized materials [31].

In the (GNPs + CNTs) sample, the D band, G band, and the 2D band were found at 1341 cm^−1^, 1585 cm^−1,^ and 2679 cm^−1^, respectively (Figure 5b). It has been observed that the intensity of the G band was higher than the D band in GNPs; however, the intensity of D was higher than the G band in (GNPs + CNTs) sample [31]. The spectra obtained thus confirmed the presence of GNPs and CNTs in the coatings. The I_D_/I_G_ ratio and I_2D_/I_G_ ratio of samples with 5% (*w*/*v*) GNPs were determined as 0.12 and 0.38, respectively. The I_D_/I_G_ ratio indicates the presence of few defects and I_2D_/I_G_ ratio points to multilayers in GNPs, respectively [14]. Likewise, the I_D_/I_G_ ratios of samples with 5% (*w*/*v*) (GNPs + CNTs) were determined as 1.05 and 0.26, respectively. The I_D_/I_G_ ratio of GNPs is smaller than that of (GNPs + CNTs) and additionally, the intensity of the 2D band is higher for GNPs compared to (GNPs + CNTs). This fact indicates a higher crystalline structure in GNPs than CNTs [29,31]. It is important to stress the very high difference in the I_D_/I_2D_ ratio between GNPs and (GNPs + CNTs) samples (0.33 and 4.00, respectively) which indicates the higher amorphous component in the (GNPs + CNTs) sample due to the carbon originally present in the raw material [32].

### 3.3. Thermogravimetric Analysis

**Figure 6:**
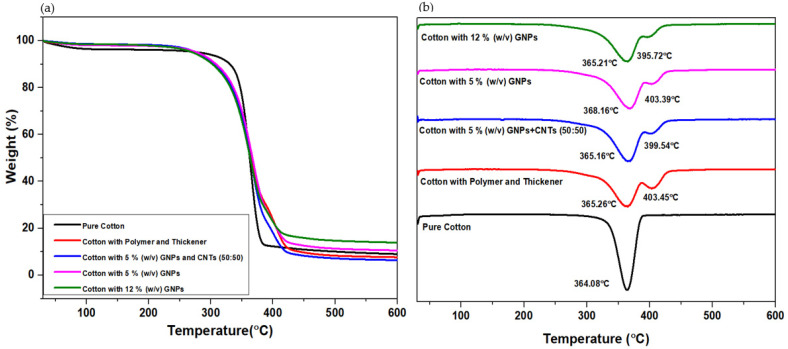
(**a**). TGA weight-loss graphs; (**b**). DTG graphs.

The thermal behavior of the pure cotton woven fabric coated with polymer and carbon-based nanoparticles was investigated using TGA under an argon gas environment. The TGA spectra are shown in Figure 6a and the derivative thermogravimetry graphs (DTG) in Figure 6b. The maximum decomposition temperature of all the samples was in the range of 250 °C to 450 °C, which can be attributed to the rapid depolymerization reactions of hemicellulose, lignin, and polyurethane matrix [13]. All the samples have shown some water-weight loss near 100 °C, which is due to the hygroscopic nature of the cotton fabric material. The weight loss in pure cotton occurred in a single step, whereas the degradation of the cellulose starts at 290 °C and ends at around 390 °C with a peak temperature of 364 °C. Similar observations were reported in a previous work [33]. The sample coated with the polymers and polyurethane-based thickener and with the carbon-based paste has shown a slightly higher weight loss, occurring in two stages. The peak at approximately 364 °C corresponds to the decomposition of hemicellulose and lignin, whereas the second peak at approximately 400 °C corresponds to the polyurethane scission and depolymerization of soft segments (polyols) as previously observed [34,35]. The residual masses of the cotton, cotton with polymer and thickener, cotton coated with 5% (*w*/*v*) GNPs, 12% (*w*/*v*) GNPs and 5% (*w*/*v*) (CNTs + GNPs) were found to be 9.0%, 7.6%, 10.6%, 13.9%, and 6.4%, respectively. The weight loss is clearly inversely proportional to the percentages of GNPs in the coated samples. The mass loss (%) that occurred in the second stage of the 5% (*w*/*v*) GNPs and 12% (*w*/*v*) GNPs was 18.53% and 17.98%, respectively. The decrement in the char residues indicates slow carbon decomposition after 400 °C. Similar observations were also reported in a previous work [36].

In the presence of the polyurethane-based polymer and thickener, the degradation started earlier. However, the carbonaceous coatings did not influence the decomposition temperature of the cotton. Moreover, the addition of CNTs, which reduces the total amount of GNPs in the coated sample, seems to not deteriorate the thermal performance of the coated textile. TGA analysis provides information about the temperature that the sample can withstand, and this information plays a crucial role in understanding their areas of application [37]. It can be understood that these coated textiles cannot be used for temperatures higher than cellulose degradation, but are suitable for medium-temperature applications.

### 3.4. Field-Emission Scanning Microscopy

**Figure 7:**
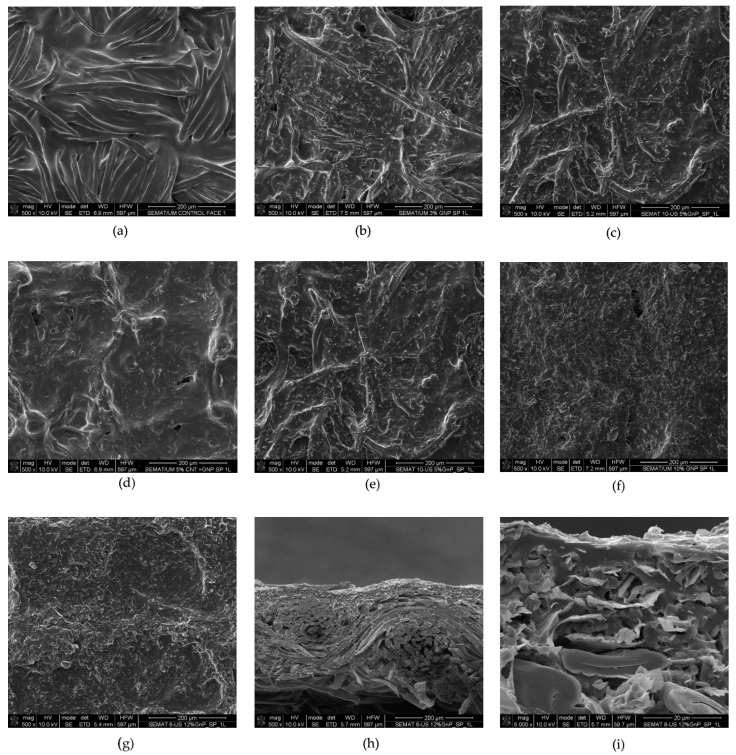
(**a**). Cotton with polymer; (**b**). 3% (*w*/*v*) GNPs; (**c**). 5% (*w*/*v*) GNPs; (**d**). 5% (*w*/*v*) CNTs + GNPs; (**e**). 7% (*w*/*v*) GNPs; (**f**). 10% (*w*/*v*) GNPs; (**g**). 12% (*w*/*v*) GNPs; (**h**). Cross section 12% (*w*/*v*) GNPs 1000×; (**i**). Cross section 12% (*w*/*v*) GNPs 5000×.

The dispersion of the GNPs and the formation of conductive networks can be analyzed by the FESEM technique. The morphology of the coated samples is displayed in Figure 7, with Figure 7a corresponding to the cotton with polymer and Figure 7b–g to the samples with various % (*w*/*v*) of GNPs. Among them, Figure 7d represents the samples with the combination of GNPs and CNTs, and additionally, Figure 7h–i represents the cross sections of the samples. From the images, a laminar layer is seen in the images of the coated samples, thus indicating that the pastes were successfully coated on the cotton fabrics. It can be observed from the images that GNPs were present in all the samples in a higher density for the samples with larger % (*w*/*v*) of GNPs, proving the successful coating process. Figure 7a–g display the fibers coated with the polymer and also the distribution of GNPs on the sample surface. Furthermore, the formation of networks for the improvement of electrical conductivity can be observed in Figure 7b–g, with evident network improvement with the increment of % (*w*/*v*) of GNPs. It can be correlated to the improvement of electrical conductivity of the coated samples with an increase in GNP concentration. It was seen in Figure 7g that the concentration of GNPs was more in comparison with Figure 7b–f. By observing Figure 7h, the coating of the polymer with GNPs on the cotton fabric was visible. The images also confirmed good adhesion between the matrix with GNPs and the fibers of the cotton fabric. Moreover, the polymer provided good anchorage for the GNPs to place themselves on the fabric and provide good pathways for the conduction of electrons. The interactions between the GNPs can be seen from the magnified FESEM image in Figure 7i. Additionally, sheet-like layers of GNPs were arranged in parallel to the cotton surface and also interpenetrated into adjacent layers. The orientation can be due to the shear force applied by the roller. The higher GNP concentration contributes to the overlapping of the GNPs and formation of the good conductive network, which corroborates the highest electrical conductivity and highest surface temperature achieved [36].

### 3.5. Electrical Conductivity

Some factors determine the electrical conductivity of a material, such as the type, aspect ratio, morphology, and conductivity of nanoparticles, but also the mixing method used, which determines the shear force on the nanoparticles. In particular, little agglomerates assist in improving electrical conductivity as they can create a connection with the neighboring nanoparticles. Moreover, it has been reported that a higher aspect ratio helps to achieve the percolation threshold at lower weight concentrations and the greater surface to volume of filler leads to better interparticle contact [38].

**Figure 8:**
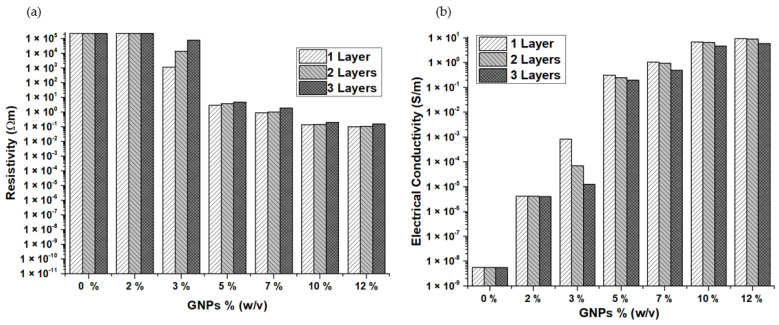
(**a**). Electrical resistivity of cotton fabrics coated with GNPs; (**b**). Electrical conductivity of cotton fabrics coated with GNPs.

Electrical resistivity and conductivity were measured for the pure cotton and samples coated with different weight fractions of nanoparticles and different layers on the cotton fabric. The pristine cotton has a very high resistivity value in the order of 10^7^ Ωm, which results in a very low conductivity value in the order of 10^−8^ S/m. The resistivity and conductivity values of the samples are provided in Tables S2–S4 in Supplementary Materials. It was observed from Figure 8a,b that the electrical resistivity was reduced, and on the contrary, the electrical conductivity was enhanced, with the addition of GNPs. Additionally, the conductivity continued to improve with the increase in the GNP concentration in the paste, which agrees with observations reported in other works [19,20,21,24]. This improvement can be because the incorporation of GNPs has created a path for the electricity flow, and increasing the concentration of the nanoparticles might have resulted in the formation of a better conductive network, which eased the flow of electrons, leading to the linear rise in the electrical conductivity [21]. The number of the layers also influenced the electrical conductivity; in all the cases, it was evident that the samples with only one layer (0.04 mm to 0.05 mm) displayed the largest value, followed by the second (0.06 mm to 0.08 mm) and third layers (0.08 to 0.11 mm). The increase in thickness of the sample has increased the resistivity, and this tendency was also reported in [26], where initially, the resistivity decreased with the increase in thickness of the film and after reaching a certain value of thickness, resistivity tends to rise again. In the present work, it is believed that the minimum film thickness obtained was already more than the optimized value, and hence resistivity increased with an increase in thickness, and electrical conductivity reduced. Hence, it can be understood that the thickness of the coating can also be one of the prominent factors that impact the conductivity of the samples.

**Figure 9:**
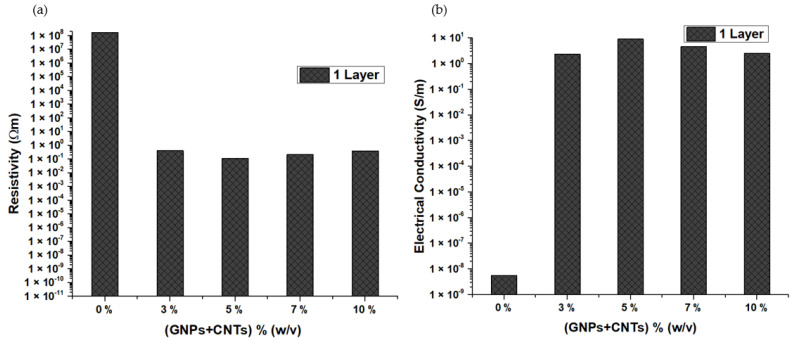
(**a**). Electrical resistivity of cotton fabrics coated with 1 layer of GNPs and CNTs; (**b**). Electrical conductivity of cotton fabrics coated with 1 layer of GNPs and CNTs.

**Figure 10:**
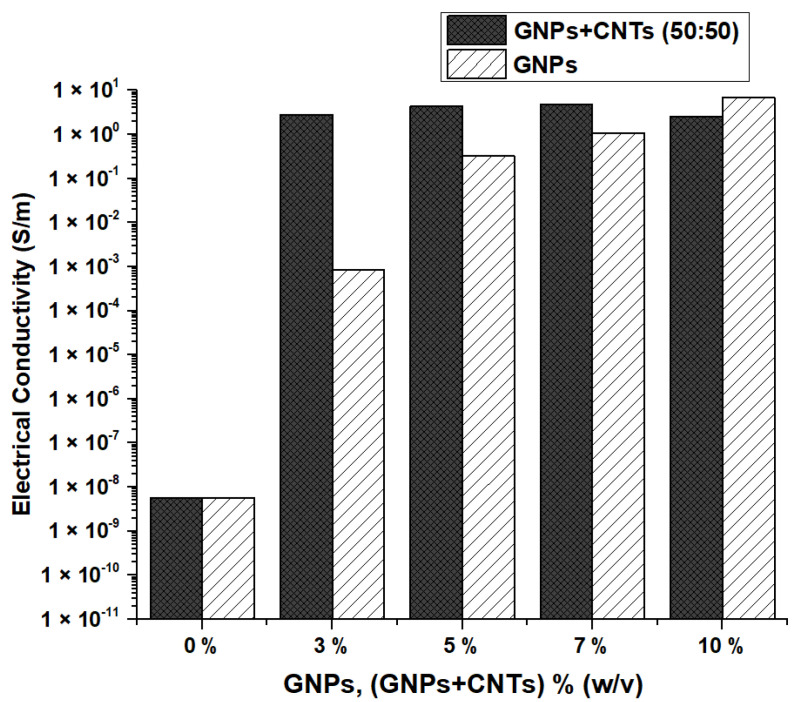
Comparison of electrical conductivity of cotton fabrics coated with 1 layer (GNPs + CNTs and GNPs only).

CNTs and GNPs are one-dimensional and two-dimensional materials, respectively. GNPs can be hybridized with low-cost CNTs to take the advantage of variation in their morphologies. Long tubular CNTs can act as a bridge between the high-aspect-ratio GNPs and facilitate the creation of an effective conductive network [24,39]. The results from Figure 9a,b suggest that the resistivity was decreased and the conductivity was enhanced with the addition of CNTs to the GNPs in the ratio of 50:50. The conductivity improved proportionally until 5% (*w*/*v*) and declined thereafter, which can be attributed to reaching the optimum concentration. From Figure 9b and Figure 10, it can be observed that the addition of CNTs has a significant improvement. The samples with 5% (*w*/*v*) (CNTs + GNPs) have attained a larger conductivity value (9.02 ± 6.62 × 10^−2^ S/m) than the ones with 10% (*w*/*v*) GNPs (6.92 ± 1.99 × 10^−1^ S/m) and approximately the same values when compared with 12% (*w*/*v*) GNPs (9.39 ± 1.28 × 10^−1^ S/m). Hence, it was evident that a good conductive network can be formed with the usage of CNTs and GNPs together in the insulating polymer matrix, and also supports the observations from previous reports [25,39]. It was clear from the results that using GNPs and CNTs simultaneously was an effective strategy to obtain higher conductivity values with lower filler concentrations [39]. In addition, these values were good enough for the coated samples to be used in some applications such as sensors, conductive thin films, and EMI shielding [25].

### 3.6. Joule Heating

**Figure 11:**
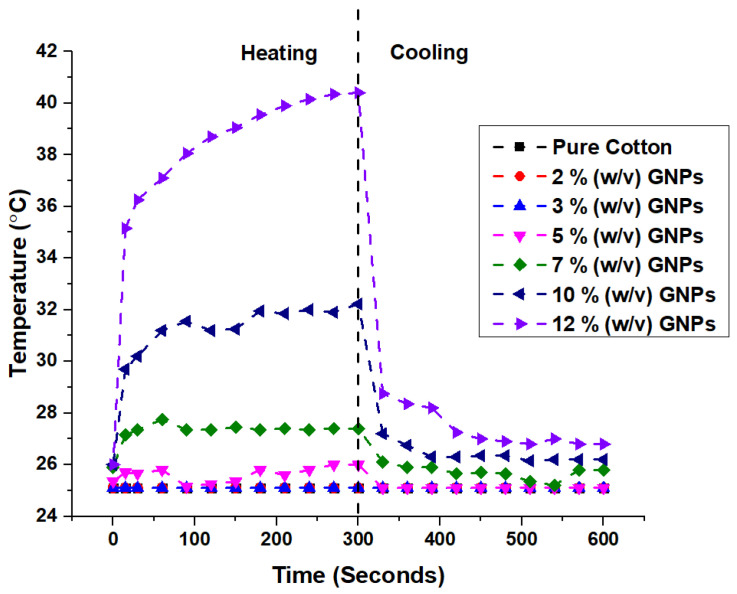
Heating of cotton fabrics coated with 1 Layer GNPs.

Certain resistivity and conductivity values are needed to obtain a heating effect according to Joule’s Law. For the generation of active heat in a fabric, the coating of a paste made of an active material with good conductivity mixed with an insulating polymer is one of the methodologies [1]. In this study, the heating effect of the coated samples was investigated by applying 12 V, in particular to be able to use them in automotive applications. Only the samples with the highest values of conductivity in each concentration were selected for assessment of the heating effect, i.e., samples with 1 layer of coating of GNPs and (GNPs + CNTs). A voltage of 12 V was applied to the coated samples, and the behavior of the rise and fall in surface temperature was observed and recorded by a thermography camera, as shown in Figure 4. Figure 11 shows the temperature increase when 12 V was applied for 300 s and 300 s after cutting off the power to the samples. The results show that the temperature rose with increased GNP concentration, with the maximum average temperature (40.4 °C) after 300 s being obtained for the sample with 12% (*w*/*v*) GNPs. Temperature time-dependent curves (Figure 11) reveal that there was a sudden increase in temperature for the first 15 s and then the temperature slightly stabilized, reaching the maximum temperature. For instance, the sample with 12% (*w*/*v*) GNPs reached 35.15 °C, within the first 15 s, and subsequently, the temperature rose to 40.4 °C in the next 285 s. After the current supply was turned off, the temperature drop was also very quick and it dropped down to 28.75 °C in 30 s.

**Figure 12:**
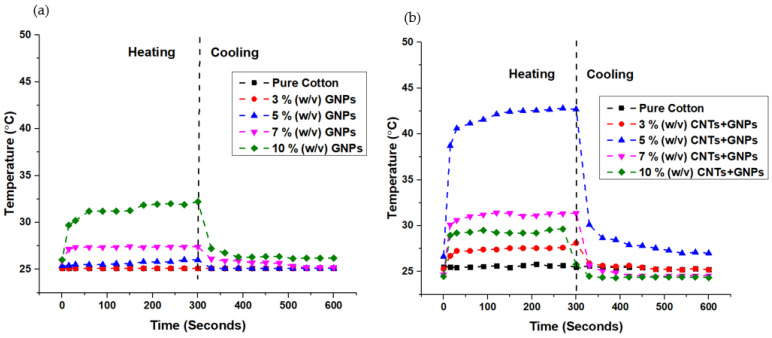
(**a**). Heating of cotton fabrics coated with 1 layer GNPs (selected % (*w*/*v*)); (**b**). Heating of cotton fabrics coated with 1 layer GNPs + CNTs (selected % (*w*/*v*)).

**Figure 13:**
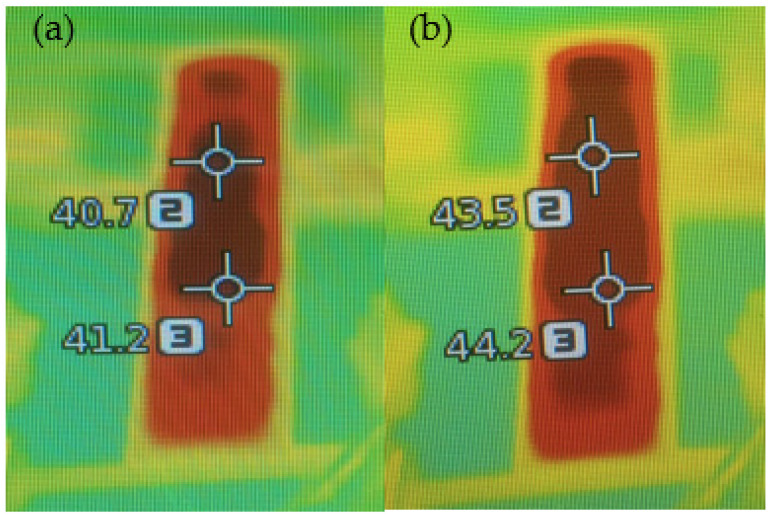
Thermography image of coated cotton fabrics 1 Layer (**a**). 12% (*w*/*v*) GNPs; (**b**). 5% (*w*/*v*) GNPs + CNTs.

When CNTs were mixed with GNPs, temperatures improved with concentration, but only until 5% (*w*/*v*), with lower temperatures achieved above that concentration (Figure 12b). This can be explained by the saturation of the conductivity value, as mentioned previously, corroborating the previous results. The sample with a 5% (*w*/*v*) (GNP + CNT) concentration reached the highest average temperature of 42.7 °C. It followed the same trend of the sample with GNPs only, reaching 38.7 °C in the first 15 s and 42.7 °C after 300 s. According to Figure 12a,b, it was evident that samples with 5% (*w*/*v*) (GNPs + CNTs) reached higher temperatures than the samples with GNPs only at the same concentration. This behavior can be attributed to a good connection attained between long tubular CNTs and flake GNPs, in comparison with samples with GNPs only, thus forming a proper conductive network for conductivity yet maintaining sufficient resistivity. Figure 13a,b display the thermographic images of the 12% (*w*/*v*) GNPs and 5% (*w*/*v*) (CNTs + GNPs) samples that showed the highest temperatures after 5 min by applying 12 V.

Figure 13a, b displays the thermographic images of the 12% (*w*/*v*) GNPs and 5% (*w*/*v*) (CNTs + GNPs) samples, which showed the highest temperatures after 5 min of applying 12 V.

## 4. Conclusions

The functionalization of cotton-woven fabrics by screen printing with carbon-based nanomaterials has created electrically conductive textiles able to be used as active heat-dissipation films. The type of nanofillers, their weight fraction, and thickness are the influencing factors in the enhancement of electrical conductivity and heat dissipation of the produced films. The sample with 12% (*w/v*) GNPs resulted in the highest value of electrical conductivity (9.39 ± 1.28 × 10^−1^ S/m) and the hybridization of CNTs with GNP samples with 5% (*w*/*v*) (CNTs + GNPs) displayed nearly the same electrical conductivity (9.02 ± 6.62 × 10^−2^ S/m). Hence, it can be concluded that the addition of CNTs can improve electrical conductivity, avoiding the usage of large amounts of GNPs, which can be cost-effective. A similar pattern was also observed in terms of the heat-dissipating ability. The highest temperature on the sample surface (42.7 °C) was obtained for the sample coated with 5% (*w*/*v*) (CNTs + GNPs), whereas 40.4 °C was obtained for the sample with 12% (*w*/*v*) GNPs. These coated samples can potentially find their applications in smart textiles to be used in automobiles, aerospace, and construction industries with medium temperatures. Mechanical durability, accelerated aging, UV, flammability tests, and oxidation tests must be performed in the future in order to use these coated fabrics for heating or other applications within the automotive industry. For smart textiles and wearable applications, flexibility, stretchability, and washing cycles should be carried out. For some of these applications, a protection layer might be needed to ensure a better stability to wash cycles but also to avoid the inhalation of these carbon-based nanomaterials or their direct contact with skin.

## Figures and Tables

**Table 1:** Mechanical and functional properties of graphene [17].

Mechanical Properties		Electrical Properties		Other Properties	
Young’s Modulus	1 TPa	Electrical Conductivity	104 S/cm	Thermal Conductivity	5300 W/mK
Fracture Strength	130 GPa	Electron Mobility	250,000 cm^2^/V.s	Specific Surface Area	2630 m^2^/g
				Optical Transmittance	97.7%

**Table 2:** Thickness of the films on the coated samples (mm).

*w*/*v* %	GNPs Samples			GNPs + CNTs Samples
	1 Layer	2 Layers	3 Layers	1 Layer
2	0.04	0.06	0.08	-
3	0.04	0.07	0.08	0.05
5	0.04	0.06	0.09	0.05
7	0.04	0.07	0.10	0.04
10	0.05	0.08	0.11	0.04
12	0.05	0.08	0.11	-

## Data Availability

Data is contained in supplementary material.

## Supplementary Materials

The following supporting information can be downloaded at: www.mdpi.com/article/10.3390/ma15124323/s1, Table S1: Technical data of the materials, Table S2: Electrical resistivity of various samples coated with GNPs, Table S3: Conductivity of various samples coated with GNPs, Table S4: Electrical resistivity and conductivity of various samples coated with GNPs + CNTs (50:50).
