# Supplementary material for: Joule-Heating Effect of Thin Films with Carbon-Based Nanomaterials
Source: Materials (Basel). 2022 Jun 18;15(12):4323. doi: 10.3390/ma15124323 (PMC9230175; doi:10.3390/ma15124323)
Supplement: Supplementary file 1 [file materials-15-04323-s001.zip › materials-1766782-supplementary.pdf]

materials

Supplementary Materials

Joule Heating Effect of Thin Films with Carbon-Based Nanomaterials

Usha Kiran Sanivada [1,2,3]*, Dina Esteves [1,3], Luisa M.Arruda[1,3], Carla Silva [4], Inês P. Moreira [1,3] and Raul Fangueiro [1,3]

1 Fibrenamics – Institute of Innovation in Fiber-Based Materials and Composites, Azurém Campus, 4800-058, Guimarães, Portugal
2 Mechanical Engineering and Resources Sustainability Centre (MEtRICS), University of Minho, Azurém Campus, 4800-058, Guimarães, Portugal
3 Centre for Textile Science and Technology (2C2T), University of Minho, Azurém Campus, 4800-058, Guimarães, Portugal
4 Simoldes Plastics, Research & Innovation, Rua Comendador António da Silva Rodrigues 165, Oliveira de Azeméis, Portugal

* Correspondence: ushakiran.sanivada@gmail.com (U.K.S); rfangueiro@dem.uminho.pt (R.F.)

Table S1. Technical data of the materials

Material	Technical Specifications	Supplier Details
Edolan CM	Aqueous aliphatic polycarbonate polyether polyurethane dispersion	Tanatex Chemicals, Netherlands
	Viscosity at 20°C ≤ 90 s (ISO 2431, flow cup 4 mm)	
	Density: 1.1 g/cm³	
	Ionicity: Anionic	
	White	
Tanapur EP 3061	Aqueous preparation of an aliphatic polyether polyurethane	Tanatex Chemicals, Netherlands
	Density: 1.0-1.1 g/cm³	
	Ionicity: Slightly cationic	
	Light Yellow, Yellow clear liquid	
Thickener A 02	Polyurethane preparation	Tanatex Chemicals, Netherlands
	Viscosity at 23°C: 5000-10000 mPa.s	
	Density: 1.19 g/cm³	
	Ionicity: Nonionic	
	Yellowish liquid	
GNPs	Thickness: 3 and 10 nm	Graphenest, Portugal
	Planar size: 0.5 to 0.2 µm	
	Surface Area: 150 m²/g	
CNTs	Multi-Walled Carbon Nanotubes	Iolitec Ionic Technologies, Germany
	Length: ~1.5 µm	
	90% C Purity Industrial Grade	
Cotton Fabric	Weft density = 26/cm², Warp density = 33/cm²	Lameirinho, Portugal
	GSM= 119.68 g/m²	

Table S2. Electrical resistivity of various samples coated with GNPs

GNPs % (w/v)	Electrical Resistivity (Ωm)		
	1 Layer	2 Layers	3 Layers
0	8.80E+07 ± 0.00E+00	8.80E+07 ± 0.00E+00	8.80E+07 ± 0.00E+00
2	2.24E+05 ± 9.80E+03	2.40E+05 ± 0.00E+00	2.40E+05 ± 0.00E+00
3	1.12E+03 ± 1.50E+02	1.40E+04 ± 0.00E+00	8.00E+04 ± 0.00E+00
5	3.11E+00 ± 1.85E-02	3.94E+00 ± 1.39E-01	4.97E+00 ± 2.18E-01
7	9.62E-01 ± 8.21E-02	1.06E+00 ± 4.64E-02	2.04E+00 ± 2.36E-01
10	1.45E-01 ± 4.39E-03	1.53E-01 ± 7.65E-03	2.09E-01 ± 8.33E-03
12	1.07E-01 ± 1.09E-03	1.11E-01 ± 1.83E-03	1.67E-01 ± 3.03E-03

Table S3. Conductivity of various samples coated with GNPs

GNPs % (w/v)	Electrical Conductivity (S/m)		
	1 Layer	2 Layers	3 Layers
0	1.14E-08 ± 0.00E+00	1.14E-08 ± 0.00E+00	1.14E-08 ± 0.00E+00
2	4.56E-06 ± 2.04E-07	4.17E-06 ± 0.00E+00	4.17E-06 ± 0.00E+00
3	9.58E-04 ± 1.25E-04	7.14E-05 ± 0.00E+00	1.25E-05 ± 0.00E+00
5	3.22E-01± 1.91E-03	2.55E-01 ± 9.31E-03	2.03E-01 ± 8.31E-03
7	1.06E+00 ± 7.16E-02	9.48E-01 ± 3.81E-02	5.08E-01 ± 4.29E-02
10	6.92E+00 ± 1.99E-01	6.57E+00 ± 2.90E-01	4.82E+00 ± 1.79E-01
12	9.39E+00 ± 1.28E-01	9.04E+00 ± 1.35E-01	6.01E+00 ± 1.13E-01

Table S4. Electrical resistivity and conductivity of various samples coated with GNPs + CNTs (50:50)

GNPs % (w/v)	Electrical Resistivity (Ωm)	Electrical Conductivity (S/m)
	1 Layer	1 Layer
0	8.80E+07 ± 0.00E+00	1.14E-08 ± 0.00E+00
3	4.23E-01 ± 4.02E-03	2.37E+00 ± 2.05E-02
5	1.11E-01 ± 9.46E-04	9.02E+00 ± 6.62E-02
7	2.11E-01± 1.12E-03	4.73E+00 ± 3.07E-02
10	3.96E-01± 6.80E-03	2.52E+00 ± 5.00E-02
